# Is an immune reaction required for malignant transformation and cancer growth?

**DOI:** 10.1007/s00262-012-1233-5

**Published:** 2012-05-18

**Authors:** Richmond T. Prehn, Liisa M. Prehn

**Affiliations:** Pathology Department, University of Washington, Seattle, WA USA

**Keywords:** Tumor antigens, Immune surveillance, Immune stimulation, Skin papillomas

## Abstract

Increasing evidence has shown that probably all malignant mouse cells, even those of spontaneous sporadic cancers, are endowed with tumor-specific antigens. Stimulation of cancer growth, rather than inhibition by the immune reaction, is seemingly the prevalent effect in the animal of origin (the autochthonous animal). Small initial dosages of even strong tumor antigens tend to produce stimulatory immune reactions rather than tumor inhibition in any animal. Thus, an immune response at a low level may be an essential growth-driving feature of nascent cancers, and this may be why all cancers apparently have tumor-specific antigens. Inasmuch as a low level of immunity is stimulatory to tumor growth while larger dosages are inhibitory, immuno-selection via this low response may tend to keep the antitumor immune reaction weak and at a nearly maximal stimulatory level throughout most of a tumor’s existence. These facts suggest that both suppression of tumor immunity and a heightened immune reaction might each be therapeutic although very contrasting modalities.

## Review

### Purpose and methods

We decided to reexamine the data concerning the influence of the host’s immune capacity on papilloma incidence and progression that had been obtained in our laboratory by two students, Ed Andrews and the late Marc Lappé, prior to 1972; that is, before the immuno-stimulation hypothesis was contemplated. At that time, the major question in our minds was how much substance the dominant theory of immuno-surveillance of carcinogenesis might actually have and to what extent immunity played a role in cancer resistance. Our laboratory’s work in 1967–1971 was, in part, designed to study the role of the immune reaction on papilloma production and progression in mouse skin that had been initiated with 3-methylcholanthrene (MCA) and promoted by either isotopic syngeneic or allogeneic transplantation of the initiated skins. This method of promotion was used with the thought that there might be less complication from systemic MCA [1].

In the Lappé work [1], the mice, prior to receiving syngeneic MCA-initiated skins, were immuno-modulated by an immuno-stimulant or immuno-suppressed by radiation alone or radiation combined with thymectomy. The results were as expected according to the surveillance hypothesis; that is, papilloma incidence was indirectly proportional to the degree of immuno-competency. All malignancies arose via progression in preexisting papillomas. The percentage of papillomas becoming malignant in each successive 40-day observation period was about 13 %, regardless of the treatment group, for a total of 44 transformations among the 369 papillomas available for study. Thus, at least over the range examined, variations in the level of immune capacity had no effect upon the likelihood of a transformation in any papilloma, but transformation was directly associated with the incidence of papillomas.

By way of contrast, in the Andrews paper, maximal immuno-suppression was attempted in mice bearing allogeneic MCA-initiated skin grafts [2]; the results were not as anticipated. Papilloma regressions occurred, seemingly unaffected by the lack of immunity. A total of 81 papillomas were available for at least 40 days of observation, which, according to the Lappé data, was expected to yield about ten transformants. There were no malignant transformations; transformation seemed to be dependent upon the existence of the missing immune capacity.

### Discussion

The first question is the extent to which these old data are compatible with the more recently devised immuno-stimulation hypothesis of oncogenesis [3–5]. This alternative to the immuno-surveillance theory had its first solid foundation in the 1972 paper that showed, in radiated/thymectomized mice, that specifically immune spleen cells admixed with tumor cells could either stimulate or inhibit the growth of a syngeneic tumor implant depending only upon the ratio of immune cells to tumor cells; small ratios stimulated the growths of the admixed tumor cells, while larger ratios were inhibitory [6]. Spleen cells harvested from mice immunized against a different tumor had no effect different from the effect of spleen cells obtained from non-immunized controls.

**Fig. 1:**
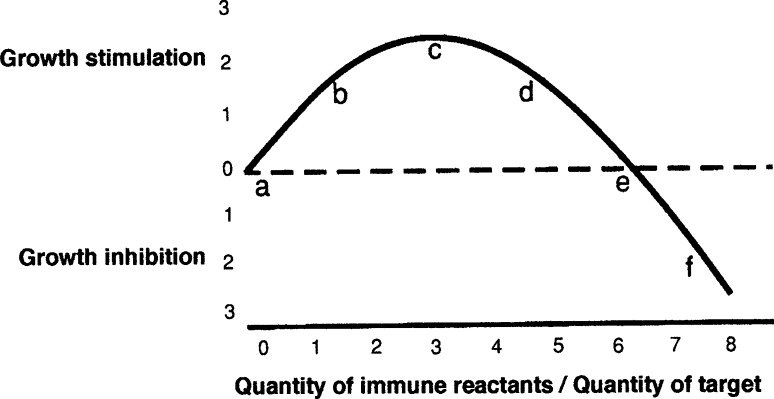
The IRC or immune reaction curve. Idealized depiction of the data from [6] showing the shape of the immune reaction curve or IRC. The *letters* and *numerals* are arbitrary aids to discussion

It is clear from inspection of the IRC (Fig. 1) that the gross data from the Lappé work are consistent with both the immuno-surveillance and the immuno-stimulation theories of tumor origin. In the surveillance theory, the transformants would all lie somewhere on the slope near or to the right of “e” on the IRC (Fig. 1) and be inhibited almost at their inception by an immune response; in the stimulation theory, the transformants would all lie to the left of “c” where an increase in immunity (a move to the right) would increase rather than decrease their growth.

In the Lappé publication, it was pointed out that a twofold increment in malignancies correlated with a twofold increment in papilloma days at risk, regardless of the immuno-competencies of the hosts. In other words, papillomas were not selected either positively or negatively by the immune response [1]. All existing papillomas had an equal opportunity for malignant transformation in any given 40-day time period. This suggests that the higher incidence of papillomas in immuno-suppressed hosts was probably unrelated to any specific immunogenicity of the transformants. Therefore, the higher incidence of papillomas in the immuno-suppressed mice may have been caused by some environmental change (perhaps related to wound healing) that had no direct relationship to any specific anticarcinoma immunity. Specific carcinoma immunogenicity apparently began with the malignant transformants, not in the preceding papillomas.

The results obtained by Andrews are at odds with the immune surveillance hypothesis. His work differed from that of Lappé only in the greater degree of immuno-suppression and the use of allogeneic rather than syngeneic skin. The work seems to show that malignant transformation fails to occur in the absence of a demonstrable immune capacity. The mice in the Andrews work were very much more immuno-suppressed than were those in the Lappé work, and no immunity could be detected by several different procedures including the survival of the allogeneic skin grafts themselves. Thus, the Andrews system was presumably very near “a” on the IRC (Fig. 1), in contrast to the Lappé papillomas, which, even in the immuno-suppressed groups, were further to the right [2].

The regression of papillomas (80 %) in the work of Andrews suggests, as did the work of Lappé, that papilloma regression was not a result of antipapilloma immunity. However, the lack of any transformation suggests that transformation may require an immune reaction, a conclusion that needs confirmation, not only because of its importance, but also owing to the limited extent of the data and because extreme immuno-suppression might have compromised the health of the mice.

It seems that all or perhaps almost all spontaneous, sporadic cancers, when transplanted, engender a tumor-stimulating immunity compatible with a very low immune reaction (Fig. 1a–d), and none engenders an inhibitory reaction [7]. This recent work [7] confirms the stimulation that was observed by Hewitt et al. and which stimulation those authors interpreted as an absence of immunity [8]. Since all immune reactions presumably begin small before they can grow large, it is natural to hypothesize that the initial immune reaction is always stimulatory rather than inhibitory to oncogenesis and to speculate that active immunity may be necessary for at least the initial growths of most or perhaps of all cancers.

These considerations, especially in combination with the Andrews observations, seem to make the case for the stimulation hypothesis. However, it might be suggested, in support of the surveillance idea, that the high immunogenicity of tumors induced by higher dosages of MCA might cause incipient tumors to lie far to the right of “c” on the IRC (Fig. 1). Such a conjecture is probably falsified by the established fact, already mentioned, that even highly immunogenic de novo tumors do not provoke, by their growth in the original host, an inhibitory immunity. Inhibitory immunity can be invoked in the animal of origin by repeated subsequent tumor inoculations [9], but the original growth of the tumor in the primary host produces only a low level of immunity that is probably stimulatory to the tumor [10–12]. This small stimulatory degree of immunity is apparently caused, in some unknown way, by the small size of the initial antigen exposure [13]. Any apparent discrepancies between the Lappé and Andrews results can be reconciled by their different locations on the IRC (Fig. 1).

### Possible correlates

It remains for further investigation to discover the natural history of cancer immunity; does the immune reaction remain low and stimulatory after the tumor’s incipiency or does it grow with time to inhibitory levels? Certainly, the rarity of spontaneous regression suggests that an immune inhibition of cancer growth, if the immunity occurs naturally, is seldom very effective. However, as already suggested, there is evidence that cancer-specific immunity can be induced even in the autologous animal by subsequent massive immunizations [9].

The Kaposi sarcoma, which is common in AIDS patients, may illuminate the role of antitumor immunity over time. This lesion sometimes “flares” during anti-AIDS therapy; apparently, the lesion grows best when the immune competency of the patient is impaired, but not too impaired [14]. A likely implication may be that even an established but untransplanted viral tumor, such as the Kaposi sarcoma, remains dependent upon a long continuing, stimulatory, low level immune reaction.

Furthermore, as already mentioned, the initial growth of a chemically induced tumor in the autologous host renders that host resistant to subsequent attempts to induce immune inhibition of the growth of an inoculum of the same tumor; the result of such a challenge may, in actuality, be some degree of overt stimulation rather than inhibition [10–12]. The lack of inhibitory immunity apparently continues for sometime, in the animal of origin, after the tumor’s excision. This conclusion may be supported by the observation that progression to a more virulent type of tumor among transplanted hamster tumors appears to depend upon a persistent stimulus by an immune reaction [15, 16] (this paper [15] was published in a now extinct journal and is very difficult to obtain. In essence, the authors report that animal tumors, transplanted to hosts of varying immuno-competence, showed that further progression was directly proportional to the host’s competence).

As in the mouse, human premalignant lesions also seem to await a random malignant transformation. Thus, the smoker accumulates with every puff an increased likelihood of malignancy; on cessation of smoking, the likelihood of malignancy remains at an elevated constant year after year [17]. This phenomenon suggests the persistence of a premalignant condition, perhaps analogous to the skin papillomas previously discussed [1].

The virus-induced hyperplastic nodules of the mouse breast seem to show a similar persistence, through time, of an unchanging incidence of random malignant transformation [18].

## Conclusions

The evidence seems consistent with the idea that the appearance of a new immunogen is a necessary trigger for oncogenesis and that immunity may continue to stimulate the growth of a cancer throughout much of the tumor’s existence. It could be that, in any untreated cancer, because of positive immune selection, whatever immunity exists may usually be near the stimulatory maximum, around “c” on the IRC.

If most cancers are, because of immune selection, found to gravitate to a position near “c” on the IRC (Fig. 1), any degree of immune alteration, either immuno-suppression or immuno-stimulation, might be expected to retard, to some degree, the growth of most malignancies. However, the incidence of some skin and lymphoreticular cancers seems to be increased rather than inhibited in immuno-suppressed allograft patients [19] suggesting that these tumors may still have been around “d” on the IRC (Fig. 1) when immuno-suppression occurred.

If these conjectures are correct, why did such a system evolve? If tolerance production was the general result of small initial doses of foreign antigen, the result would perhaps be catastrophic for resistance to infection! However, tolerance induction by a tiny initial exposure to antigen and then a continued exposure might be necessary conditions to prevent autoimmunity. Detailed speculations along these lines appear to be premature, except possibly to stimulate thought.

Note: A somewhat similar hypothesis has been advanced by Shevchenko et al. [20] (this paper is virtually unattainable in the “West”. However, we feel that it must be mentioned since it is an excellent and extensive review of the related ideas being pursued in slavic countries).
